# How Do We Taper and Discontinue Opioids in Cancer Patients? Considerations from the Activities of a Palliative Care Team at a University Hospital

**DOI:** 10.1089/pmr.2020.0105

**Published:** 2021-09-27

**Authors:** Ayumi Kihara, Kazuki Shimada, Satoru Tsuneto

**Affiliations:** ^1^ Department of Palliative Medicine, Kyoto Min-iren Asukai Hospital, Kyoto, Japan.; ^2^ Department of Palliative Medicine, Kyoto University Hospital, Kyoto, Japan.; ^3^ Department of Human Health Sciences, Graduate School of Medicine, Kyoto University, Kyoto, Japan.

**Keywords:** opioid tapering method, opioid withdrawal syndrome, physical dependence

## Abstract

***Background:*** The more the cancer treatments progress, the more the needs increase to taper and discontinue opioids in cancer patients. Furthermore, opioid dependence of cancer survivors has become a bigger problem. However, a safe opioid tapering and discontinuation method has not yet been established in cancer patients.

***Objective:*** To suggest a safe opioid tapering and discontinuation method in cancer patients.

***Design:*** We reviewed opioid type, dose, administration route, administration duration, reason for tapering and discontinuation, and presence/absence of opioid withdrawal symptoms in cancer patients whose opioids needed to be tapered and discontinued.

***Setting/Subjects:*** We recruited cancer patients referred to the palliative care team of Kyoto University Hospital-Japan whose opioids were tapered and discontinued.

***Measurements:*** Opioid withdrawal symptoms were assessed by two physicians, one nurse, and one pharmacist of palliative care team.

***Results:*** Opioids were tapered and discontinued in 25 out of 145 cancer patients (17%). Opioid withdrawal symptoms were observed in 3 of the 25 cases (12%). In withdrawal symptom cases and nonwithdrawal symptom cases, the mean maximum oral morphine-equivalent doses of opioids were 352.0 and 55.7 mg/day, and the mean administration duration of opioid were 82.3 and 28.7 days, respectively. Withdrawal symptoms occurred in patients receiving higher-dose opioids with longer administration duration and their symptoms tended to appear at approximately 10% of the maximum dose.

***Conclusions:*** We suggest that withdrawal symptoms may be prevented by using a two-stage method rather than a monotonous tapering method in cancer patients whose higher-dose opioid with longer administration duration needed to be tapered and discontinued.

## Introduction

In recent years, cancer treatment has made remarkable progress. It has led to an increase in cancer survivors and to more patients requiring tapering and discontinuation of opioids.^1^ Approximately 5%–10% of cancer survivors have severe chronic pain.^2^ There is growing concern about the long-term adverse effects of opioids and the risk of opioid misuse in cancer survivors.^3,4^

Opioid withdrawal symptoms are induced by sudden discontinuation of long-term opioid use.^5–7^ Previous studies have explored the prevention of withdrawal symptoms in patients who do not have cancer.^8–10^ Treatment of the physical dependence-related withdrawal symptoms has been developed for patients with chronic pain without cancer or opioid use disorder.^11,12^ The treatment could be applied to cancer patients who use opioids. However, there are few evidence-based studies exploring opioid tapering and discontinuation in cancer patients. The purpose of this study is to recommend a safe opioid tapering and discontinuation method for cancer patients.

## Methods

This is a retrospective chart review of patients referred to the palliative care team of Kyoto University Hospital between January and December 2015, whose pain included opioids that were discontinued within the two years preceding referral. Written informed consent was obtained from patients of case presentation. We investigated the opioid type, dose, administration route, duration of administration, reason for tapering and discontinuation, and presence/absence of opioid withdrawal symptoms. In cases of withdrawal symptoms, duration of opioid use was defined as the onset of opioid use to the onset of withdrawal symptoms. Opioid withdrawal symptoms were assessed by two physicians, one nurse, and one pharmacist of the palliative care team. We diagnosed symptoms related to opioid withdrawal if they occurred during opioid tapering, other causes could be ruled out, and they improved with opioid readministration. The estimated oral morphine-equivalent dose (MED) was calculated according to the opioid potency data in the Palliative Care Formulary.^13^

## Results

### Characteristics of the subjects

Of the 356 cancer patients referred to the palliative care team during the study period, 145 received opioids for pain. Opioids were tapered and discontinued in 25 cases (17%). Their characteristics are shown in Table 1. Opioid withdrawal symptoms were observed in 3 of the 25 cases (12%).

**Table 1.** Characteristics of Patients Discontinued Opioid (*n* = 25)

Characteristic	Nonwithdrawal syndrome cases (*n* = 22)	Withdrawal syndrome cases (*n* = 3)
Age (years)	63.7 ± 16.4	45.0 ± 3.5
Male gender (%)	40.9	33.3
Cancer diagnosis		
Gynecological	5	2
Head and neck	5	0
Lymphoma	4	0
Genitourinary	3	0
Breast	2	0
Leukemia	1	0
Lung	1	0
Melanoma	1	0
Liver	0	1
Pain causes		
Pain related to cancer	14	0
Pain related to treatment	6	2
Pain unrelated to cancer/treatment	2	1
Opioids		
Fentanyl	14	1
Oxycodone	6	2
Morphine	1	0
Tapentadol	1	0
Route of opioid administration		
Transdermal	13	0
Oral	5	1
Continuous intravenous infusion	4	2
Maximum dose of opioid		
(Oral morphine equivalent: mg/day)	55.7 ± 43.6	352.0 ± 194.0
Duration of opioid administration (days)	29.0 ± 34.5	82.0 ± 108.9

We recognized three patterns among pain etiologies. Fourteen patients had pain related to cancer, including 12 for whom cancer treatment eliminated the pain (7 had chemotherapy, 3 had radiation therapy, and 2 had surgery) and 2 cases who self-decided to discontinue. Nine patients had pain related to cancer treatment: seven had chemotherapy-related mucosal pain, two had postoperative pain, and two had infected wounds. Two patients had pain not directly related to cancer or cancer treatment (ileus and pancreatitis, respectively). Transdermal fentanyl was the most commonly chosen opioid medication for those who had difficulty taking it orally.

### Maximum oral MED and duration of administration

**FIG. 1.**
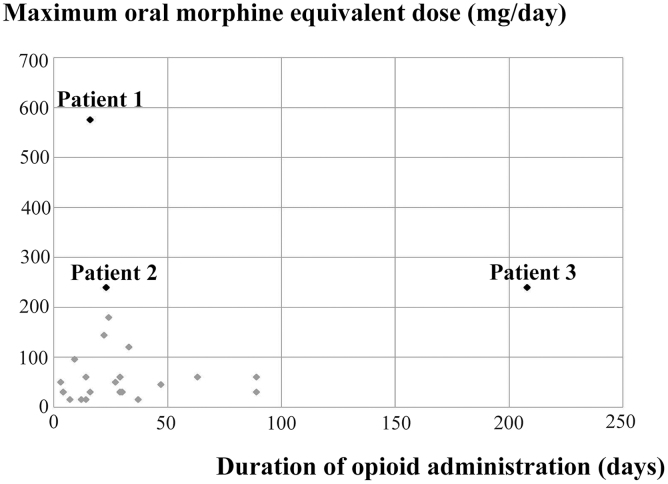
Maximum dose of opioid and duration of opioid administration in cancer patients.

The mean MED of patients with and without withdrawal symptoms was 352.0 and 55.7 mg/day, respectively. The mean duration of opioid use was 82.3 and 28.7 days, respectively (Table 1). Figure 1 shows the maximum MED and duration of opioid use for the 25 patients whose opioids were tapered and discontinued. Withdrawal symptoms occurred in three patients. The maximum MED and duration of opioid use in *Patient 1* was 576 mg/day and 18 days. For *Patient 2*, it was 240 mg/day and 23 days, and for *Patient 3*, it was 240 mg/day and 208 days.

### Case presentation

#### Patient 1

**FIG. 2.**
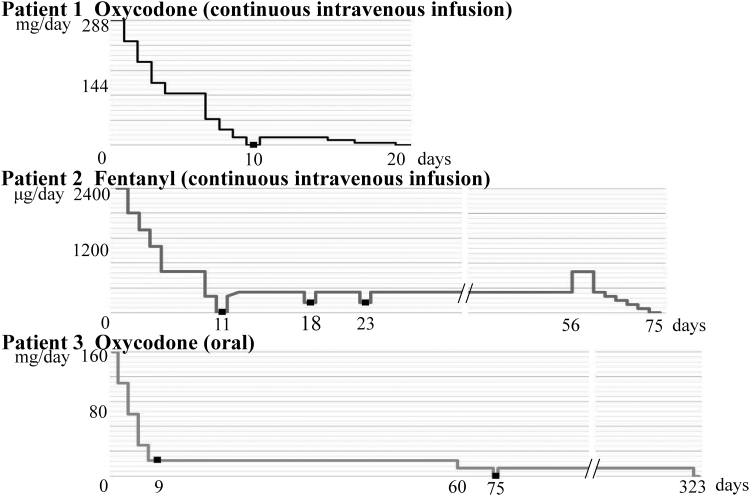
Opioid tapering line and appearance of withdrawal symptoms (■).

This was a 43-year-old woman with cervical carcinoma who received pelvic exenteration. Continuous intravenous infusion of oxycodone at 30 mg/day was started to relieve postoperative pain. The dose was increased in a stepwise way to 288 mg/day (MED 576 mg/day). The pain gradually subsided, and the oxycodone dose was reduced (Fig. 2). Continuous intravenous infusion was discontinued at 18 mg/day (6.25% of the maximum administered dose). However, six hours after discontinuation, she developed restlessness and an itching sensation in her legs. Therefore, continuous intravenous infusion of oxycodone at a dose of 18 mg/day was resumed the same day, and symptoms disappeared. The oxycodone dose was further reduced and discontinued gradually over 10 days. Subsequently, no withdrawal symptoms occurred.

#### Patient 2

This was a 43-year-old man with hepatocellular carcinoma who had undergone liver transplantation from a living donor. A year later, he developed severe acute pancreatitis with severe abdominal pain. On admission to the hospital, continuous intravenous infusion of fentanyl at 1200 μg/day was started to control abdominal pain. The fentanyl dose was increased according to pain intensity, reaching 2400 μg/day (MED 240 mg/day) after 10 days in the hospital. Administration of a pancreatic enzyme inhibitor, antibiotic, and fluid alleviated the pancreatitis and abdominal pain. Figure 2 shows that continuous fentanyl infusion was discontinued after reaching 240 μg/day (10% of the maximum dose). He subsequently developed nausea/vomiting, elevated blood pressure, and restlessness 12 hours after discontinuation. Continuous intravenous fentanyl infusion of 240 μg/day was resumed, and these symptoms disappeared. However, restlessness occurred each time the dose was reduced. Therefore, a transdermal fentanyl patch was initiated to taper the opioid more slowly. Pancreatitis with abdominal pain recurred transiently one month later. Therefore, continuous intravenous fentanyl infusion was resumed, titrated to 600 μg/day. The dose was gradually decreased daily (600, 300, 240, 180, 120, and 60 μg/day). Once the pancreatitis had improved, the fentanyl could be discontinued without causing withdrawal symptoms. The discontinuation was finally completed 64 days after the onset of withdrawal symptoms.

#### Patient 3

This was a 49-year-old woman with vulvar sarcoma who was irradiated with proton beam radiotherapy. Unfortunately, a very painful ulcer formed within the irradiation field one month after completion of treatment. Sustained-release oxycodone administration was started at 40 mg/day. The oxycodone dose was increased according to pain intensity, reaching 160 mg/day (MED 240 mg/day). The ulcer was treated with skin grafting and relieved the pain. The oxycodone dose was decreased every two days (Fig. 2). Mild restlessness occurred at 20 mg/day (12.5% of the maximum dose). Since the symptom was mild, the same dose was administered continuously and the restlessness disappeared after a few days. However, oxycodone 20 mg/day was continued for another two months before being reduced to 10 mg/day for 14 days and then discontinued. Six hours after discontinuing oxycodone, she developed cold sweats, malaise, and leg pain. Sustained-release oxycodone (10 mg/day) was restarted, and symptoms were relieved. This lasted for about five months because she was afraid of developing withdrawal symptoms. Final oxycodone discontinuation was achieved 323 days after the first onset of withdrawal symptoms.

## Discussion

World Health Organization (WHO) guidelines for the management of cancer pain have identified strategies for discontinuing in various clinical settings opioids used for chronic noncancer pain in various clinical settings that address chronic noncancer pain. The guidelines recommend that opioid be discontinued immediately without tapering for short-term opioid use (less than two weeks) if the cause of pain has been successfully treated.^14^ Physical dependence should be considered when tapering after long-term opioid use.^9,15^ However, Heishman et al. point out that withdrawal symptoms can occur after acute opioid use.^16^

In our study, nine patients showed no opioid withdrawal symptoms without opioid tapering. Their MED was 15–50 mg/day, and the duration of opioid use was 3–37 days. *Patient 1* and *Patient 2*, who had a higher maximum MED (>200 mg/day), developed opioid withdrawal symptoms. They also had a longer duration of opioid use (64 and 323 days, respectively). *Patient 2* and *Patient 3* wanted to continue opioid use because of fear of recurrence of withdrawal symptoms. It can lead to psychological dependence.^17^ Experiencing opioid withdrawal symptoms can cause anxiety about opioid tapering.^18,19^ This leads to physical and psychological dependence in some patients and may increase the risk of developing opioid use disorder. Chemical coping is often associated with anxiety.^18,20^ It is important to prevent physical and psychological dependence on opioids.

To prevent withdrawal symptoms, it is recommended that opioids be tapered by 25% until discontinuation.^5,21^ In our study, opioids were reduced by 6.25%–25% every one to two days. Berna et al. recommend reducing the dose in two stages in patients with long-term opioid use for chronic pain. In the first stage, the total dose is reduced by 10% every five to seven days until it reaches 30% of the total dose. Then the dose is reduced by 3% each week until discontinued.^22^ In our study, *Patient 1* and *Patient 2* developed withdrawal symptoms when the opioid was discontinued, and *Patient 3* developed withdrawal symptoms at 12.5% of the maximum MED. Withdrawal symptoms usually occur at about 10% or less of maximum MED. For this reason, we recommend two-stage opioid tapering in cancer patients. Our method is to first reduce 10%–20% of the total dose every two to three days until we have reduced approximately 10% of the total dose, and then 2%–3% every day thereafter. In fact, none of the cancer pain patients had withdrawal symptoms after using this method. This may prevent the onset of withdrawal symptoms in patients who have been taking higher-dose opioids for a longer duration.

Pain requires a multifaceted assessment and treatment. Palliative care should take a holistic approach to pain management and provide interdisciplinary care that balances analgesia and appropriate opioid use in the early stage of cancer treatment.^23,24^ Our interdisciplinary team (six physicians, two nurses, and two pharmacists) is working hard to improve this, and the number of the patients was small. Nevertheless, we supported the patients vigorously and continuously. In conclusion, we recommend the use of two-stage opioid tapering rather than linear tapering to prevent opioid withdrawal symptoms when a patient has been taking a higher opioid dose for a longer duration of opioid use at the start of tapering.

## Abbreviations Used

MED: morphine-equivalent dose
WHO: World Health Organization
